# SRT1720 Alleviates ANIT-Induced Cholestasis in a Mouse Model

**DOI:** 10.3389/fphar.2017.00256

**Published:** 2017-05-11

**Authors:** Linxi Yu, Xiaoxin Liu, Zihang Yuan, Xiaojiaoyang Li, Hang Yang, Ziqiao Yuan, Lixin Sun, Luyong Zhang, Zhengzhou Jiang

**Affiliations:** ^1^Jiangsu Key Laboratory of Drug Screening, China Pharmaceutical UniversityNanjing, China; ^2^Jiangsu Center for Pharmacodynamics Research and Evaluation, China Pharmaceutical UniversityNanjing, China; ^3^State Key Laboratory of Natural Medicines, China Pharmaceutical UniversityNanjing, China; ^4^Key Laboratory of Drug Quality Control and Pharmacovigilance, China Pharmaceutical University – Ministry of EducationNanjing, China

**Keywords:** SRT1720, ANTI, cholestasis, FXR, Nrf2

## Abstract

Intrahepatic cholestasis is a kind of clinical syndrome along with hepatotoxicity which caused by intrahepatic and systemic accumulations of bile acid. There are several crucial generating factors of the pathogenesis of cholestasis, such as inflammation, dysregulation of bile acid transporters and oxidative stress. SIRT1 is regarded as a class III histone deacetylase (HDAC). According to a set of researches, SIRT1 is one of the most important factors which can regulate the hepatic bile acid metabolism. SRT1720 is a kind of activator of SIRT1 which is 1000 times more potent than resveratrol, and this paper is aimed to study its protective influence on hepatotoxicity and cholestasis induced by alpha-naphthylisothiocyanate (ANIT) in mice. The findings revealed that SRT1720 treatment increased FXR and Nrf2 gene expressions to shield against hepatotoxicity and cholestasis induced by ANIT. The mRNA levels of hepatic bile acid transporters were also altered by SRT1720. Furthermore, SRT1720 enhanced the antioxidative system by increasing Nrf2, SOD, GCLc, GCLm, Nqo1, and HO-1 gene expressions. In conclusion, a protective influence could be provided by SRT1720 to cure ANIT-induced hepatotoxicity and cholestasis, which was partly through FXR and Nrf2 activations. These results indicated that SIRT1 could be regarded as a therapeutic target to cure the cholestasis.

## Introduction

Intrahepatic cholestasis is a kind of hepatic disease characterized by symptoms including systematic and intrahepatic accumulations of excessive bile acid. It may be caused by pregnancy, drugs, hormones, and inflammatory cytokines, or progressive bile duct destruction. And it increases the risk of hepatitis, liver cirrhosis, or other hepatic and gall-bladder diseases (Stone et al., 2012; Hirschfield et al., 2013; Geenes et al., 2014; Ji et al., 2014; Meng et al., 2015a). Recently, standard treatment for cholesteric diseases is limited to the use of ursodeoxycholic acid (UDCA) for primary biliary cholangitis (PBC), while there are no accepted treatments for other adult cholesteric disorders (Tanaka and Gershwin, 2017) Therefore, looking for new targets and developing new drugs for the treatment of cholestasis are necessary.

Alpha-naphthylisothiocyanate (ANIT) is a kind of chemical substance which can cause acute cholesteric liver injury in rodents. It is widely utilized because it can cause cholestasis repeatedly. A single dose of ANIT to rat can lead the impairment of bile duct epithelial cell (Dahm and Roth, 1991). The mechanisms of ANIT-induced liver injury are not entirely clarified. However, it has been proved that ANIT inhibited the expressions of bile acid transporters in mouse primary hepatocytes (Guo et al., 2014). In the liver, ANIT also significantly decreased the expressions of Mrp2 (multidrug resistance-associated protein 2), FXR (farnesoid X receptor), Ntcp (Na^+^-dependent taurocholate cotransporter), Bsep (bile salt export pump), and CYP7A1 (cholesterol 7α-hydroxylase) (Wang T. et al., 2014), and it has been reported that ANIT made bile duct epithelial cells produce substance which was regarded as an activator for neutrophils to hurt hepatocytes (Hill and Roth, 1998; Kobayashi et al., 2010). The lipid peroxidation caused by reactive oxygen species (ROS) was also related to the development of the liver injury that was induced by ANIT in mice (Kongo et al., 1999). ANIT also led to inflammatory reactions, it could increase the levels of TNF-α (tumor necrosis factor alpha) and IL-6 (interleukin-6) in rat livers (Wang T. et al., 2014). Therefore, inflammation, dysregulation of bile acid transporters and oxidative stress are important generating factors for the liver injury induced by ANIT (Ma et al., 2015).

SIRT1 is one of the important silent information regulators and it is an NAD-dependent deacetylase which can regulate a wide range of cellular processes, including inflammation, aging, and lifespan extension (Bordone et al., 2007). SIRT1 regulates various nuclear receptors and cofactors directly or indirectly, such as FXR, HNF1α, LXR, E2F1, p53, PGC-1α, and HSF1 (Gerhart-Hines et al., 2007; Hou et al., 2008; Ponugoti et al., 2010). It has been considered to be a metabolic sensor for a wide range of metabolic processes. FXR has been considered as a crucial nuclear receptor in bile acid metabolism. Hepatic SIRT1 deficiency decreased the gene expression of FXR and increased hepatic BA concentrations. It largely through lack of hepatic SIRT1 down-regulated HNF1α (hepatocyte nuclear factor 1α)-FXR signaling. HNF1α is a transcription factor which can bind to the promoter of FXR directly. Deficiency of SIRT1 decreased the recruitment of HNF1α to the promoter of FXR and decreased the FXR mRNA level along with FXR target genes such as Bsep and short heterodimer partner (Shp), as a result, transport process of phospholipids and biliary bile acid can be injured (Kemper et al., 2009; Purushotham et al., 2012; Kazgan et al., 2014). Therefore, liver SIRT1 can regulate FXR and is one of the most important factors which can influence the metabolism of hepatic bile acid, and it has been reported that SRT1720 can treat the cholesteric liver injury in a mice model of cholestasis (Kulkarni et al., 2016).

Nrf2 (nuclear factor erythroid 2-related factor 2), a transcription factor, seems to be a sensor to regulate various antioxidative stress genes expressions (Xu et al., 2008). Emerging evidence has shown that the Nrf2/ARE (antioxidant response element) antioxidant pathway is important in ANTI-induced cholestasis and liver injury (Ma et al., 2015). SIRT1 can increase Nrf2 protein expression and enhance gene expressions of its downstream genes: SOD and HO-1. Furthermore, through regulating the Nrf2 deacetylation function, SIRT1 can improve Nrf2 stability. Therefore, SIRT1 could help cells to avoid oxidative stress-induced injury through active Nrf2 and its downstream gene expressions (Xue et al., 2016).

Taken together, SIRT1 is associated with the activations of FXR and Nrf2. Therefore, we speculate that SIRT1 is a therapeutic target for the cholestasis treatment. To verify our hypothesis, SRT1720, a specific activator of SIRT1, was chosen. The compound is attached to the SIRT1 enzyme-peptide substrate composition at an allosteric site which is at the end of an amino acid with catalyst; it decreases the Michaelis constant for substrates that are acetylated. Under certain circumstances, such as when mice are obese, the compound increases the sensitive for insulin and the capacity of mitochondrion, and decreases plasma glucose level. It is 1000 times more potent than resveratrol. In fact, resveratrol is not a unique activator for SIRT1 (Milne et al., 2007; Smith et al., 2009).

Although SIRT1 could modulate bile acid metabolism, it was not confirmed whether its activator could reverse ANIT-induced cholestasis and liver injury. The aim of this study was to investigate whether oral administration of SRT1720, the activator of SIRT1, could alleviate ANIT-induced cholestasis and hepatotoxicity in mice. Additionally, the potential mechanisms would be studied *in vivo* and *in vitro* experiments.

## Materials and Methods

### Chemical Drugs

ANIT (purity >98%) has been collected from Sigma–Aldrich Co. (St Louis, MO, USA). SRT1720 was purchased from Medscheme Express Co., Ltd. (Princeton, NJ, USA). Serum alanine-transaminase (ALT), alkaline phosphatase (ALP), total bile acid (TBA), aspartate transaminase (AST), complete bilirubin (TBIL), and gamma-glutamyl transferees (γ-GGT) had been analyzed utilizing commercial kits in accordance with the protocols provided by manufacturers (Whitman Biotech, Nanjing, China). Antibodies to BSEP (sc-74500), MRP2 (sc-5770), and HNF1α (sc-135939) were provided by Santa Cruz Biotechnology Inc. (Dallas, TX, USA). Antibody to FXR (bs-12867R) was obtained from Bioss Biotechnology (Bioss, Beijing, China).

### Experimental Animals and Drug Therapy

Female C57BL/6 mice (8–9 weeks old) had been procured from SIPPR-BK Laboratory Animal Enterprise (Shanghai, China), and they were put in the 12-hour dark/light cycle. All of the mice had been sheltered in the conditions where there was not germ with constant humidity and controlled temperature (24°C ± 2°C). The mice were provided with free liquid and normal food. Previous to the tests, the mice had been acclimated to the laboratory environment for 1 week. Animal Ethics Council of China Pharmaceutical University has given permission to all animal treatment and proceedings. The tests were implemented in accordance with the Declaration of Helsinki. SRT1720 was prepared in in 0.5% CMC-Na suspension. ANIT was dissolved in olive oil. Mice had been divided into four groups casually (*n* = 6): (1) control group, in which mice were treated with 0.2% carboxymethylcellulose solution (ip) for 5 days; (2) ANIT group, in which the mice took 50 mg/kg dose of ANIT orally, at 48h before sacrifice; (3) ANIT+ SRT1720 (10 mg/kg), in which mice were given SRT1720 (10 mg/kg, ip) for five days; on the 3rd day, 4 h after SRT1720 or excipient medication, mice took ANIT (50 mg/kg) orally for 48h; (4) ANIT+SRT1720 (20 mg/kg), in which mice were treated with SRT1720 (20 mg/kg, ip) for 5 days; on the 3rd day, 4 h after SRT1720 or excipient medication, mice took ANIT (50 mg/kg) orally for 48 h. On the fifth day, 4 h after SRT1720 (10 mg/kg, 20 mg/kg) and vehicle treatment, mice were sacrificed to collect livers and blood. The protocol for the other animal experiment was as below. Mice had been divided into four groups casually (*n* = 6): (1) control group, in which mice were treated with 0.2% carboxymethylcellulose solution (ip) for 5 days; (2) ANIT group, in which the mice took 50 mg/kg dose of ANIT orally, at 24 h before sacrifice; (3) ANIT+ SRT1720 (10 mg/kg), in which mice were given SRT1720 (10 mg/kg, ip) for 5 days; on the 4th day, 4 h after SRT1720, or excipient medication, mice took ANIT (50 mg/kg) orally for 24 h; (4) ANIT+SRT1720 (20 mg/kg), in which mice were treated with SRT1720 (20 mg/kg, ip) for 5 days; on the 4th day, 4 h after SRT1720 or excipient medication, mice took ANIT (50 mg/kg) orally for 24 h. On the 5th day, 4 h after SRT1720 (10 mg/kg, 20 mg/kg) and vehicle treatment, mice were sacrificed to collect livers and blood.

The blood had been gathered in the tubes with no anticoagulation and centrifuged at room temperature to obtain serum. Livers had been put in the 10% formaldehyde solution.

### Serum and Liver Biochemistry Analysis

After sacrificed, blood of mice was centrifuged for serum and 50 mg liver tissue was collected to homogenize in RIPA buffer. ALT, ALP, AST, TBA, TBIL, and γ-GGT in mice serum were measured by corresponding commercial kits in accordance with the producer’s protocols (Whitman Biotech, Nanjing, China). Liver samples were homogenization with RIPA buffer to measure the content of bile acid. The bile acid in supernant after centrifugation had been analyzed utilizing a commercial kit (Whitman Biotech) in accordance with the protocol. The results were normalized with total protein concentrations which were evaluated by BCA protein assay Kit (Beyotime, Shanghai, China). The results obtained were the mean of six different animal livers.

### Histopathological Evaluations

After scarified, small pieces of mice livers were collected and directly put in the 10% formaldehyde solution, then inserted in paraffin and sliced to 6 μm slices. All slices were stained with H&E for necrotic and hepatocyte degeneration variations. Images were captured with the microscope (Olympus IX81). Then pathology assessments were performed by a professional pathologist (Wenxia Bai, Jiangsu Medicine Institute, Nanjing, China).

### Quantitative Real-Time Polymerase Chain Reaction

For animal experiment, 50 mg liver tissue was prepared from each mouse. TRIzol agent (Invitrogen Life Technologies, Carlsbad, USA) had been utilized to isolate entire RNA from liver. Then 2 μg of entire RNA was reversed into cDNA by Prime Script RT reagent (Takara, Osaka, Japan). QPCR had been implemented with SYBR PCR Master Mix (Takara, Osaka, Japan) with specific PCR primers (**Table 1**), and reports were generated on IQTM5 Optical System Software (Version 2.1, Bio-Rad). β-actin had been normalized as an internal control. Primers set for the HNF1α, FXR, Ntcp, Oatp1b2, Bsep, Mrp2, Mrp3, Mrp4, Cyp7A1, Shp, Nrf2, SOD, GCLc, GCLm, HO-1, and Nqo1 genes are shown in **Table 1**.

**Table 1:** The primer sequences used for real-time PCR assay in mice.

Gene	Forward primer (5′–3′)	Reverse primer (5′–3′)
HNF1α	GACCTGACCGAGTTGCCTAAT	CCGGCTCTTTCAGAATGGGT
FXR	GCTTGATGTGCTACAAAAGCTG	CGTGGTGATGGTTGAATGTCC
Ntcp	GCATGATGCCACTCCTCTTATAC	TACATAGTGTGGCCTTTTGGACT
Oatp1b2	GGGAACATGCTTCGTGGGATA	GGAGTTATGCGGACACTTCTC
Bsep	AGCAGGCTCAGCTGCATGAC	AATGGCCCGAGCAATAGCAA
Mrp2	AACTGCCTCTTCAGAATCTTA	GCCAGCCACGGAACCAGCTGCT
Mrp3	CTGGGTCCCCTGCATCTAC	GCCGTCTTGAGCCTGGATAAC
Mrp4	CATCGCGGTAACCGTCCTC	CCGCAGTTTTACTCCGCAG
Cyp7A1	CAAGAACCTGTACATGAGGGAC	CACTTCTTCAGAGGCTGCTTTC
Shp	CCCCTATCTCTCAGTACACATGG	GACCATAAGGAGGACAAAGGTCT
Nrf2	TCTTGGAGTAAGTCGAGAAGTGT	GTTGAAACTGAGCGAAAAAGGC
SOD	GCCCGCTAAGTGCTGAGTC	CCAGAAGGATAACGGATGCCA
GCLm	AGGAGCTTCGGGACTGTATCC	GGGACATGGTGCATTCCAAAA
GCLc	GGGGTGACGAGGTGGAGTA	GTTGGGGTTTGTCCTCTCCC
Nqo1	ATGGGAGGTGGTCGAATCTGA	GCCTTCCTTATACGCCAGAGATG
HO-1	AAGCCGAGAATGCTGAGTTCA	GCCGTGTAGATATGGTACAAGGA
β-actin	TATTGGCAACGAGCGGTTC	ATGCCACAGGATTCCATACCC

### Western Blot Analysis

A total protein extraction kit (KeyGEN Biotech) had been used to extract liver protein. The level of the protein was evaluated by the BCA protein assay Kit (Beyotime Biotech, Shanghai, China). Fifty microgram protein was added to the each hole of SDS-PAGE gel (10%) and then removed to PVDF membranes. After being blocked with 5% skimmed milk for 1 h, the membranes were further incubated with special antibodies (FXR, Bsep, Mrp2, HNF1α, and GAPDH) overnight at 4°C. After rinsed three times with TBST, membranes were incubated with second antibodies at room temperature for 1 h. Then Bio-Rad Imaging System (Bio-Rad, Hercules, CA, USA) was used to detect certain bands with ECL reagents (Thermo, Waltham, MA, USA).

### Immunofluorescent Staining

Cut the liver tissue into appropriate size, and fixed them on the frozen embedding mold utilizing O.T.C embedding agent. Adjusted the parameters of frozen slicer and started to cut liver tissue. The thickness of the slice is about 6 μM. Then placed slices on APES treated slides. 30 min later, the slices had been put inside formaldehyde solution (4%) for 30 min and were penetrated with Triton X-100 (0.1%) for 15 mins. Then, slices were blocking with 5% BSA for 1 h to block non-specific binding and incubated with different antibodies, placed in humidified atmosphere during the night at the temperature of 4°C. The antibodies adopted in the research were Bsep and Mrp2. The slices were then wished with phosphate-buffered saline (PBS) for three times and incubated with secondary antibodies for 1 h. Finally, DAPI had been utilized for nuclear staining. After that, images were obtained from Olympus IX81 motorized inverted fluorescence microscope and analyzed by Image-Pro plus 10.0 software.

### Isolation and Sandwich Cultivation of Mice Primary Hepatocytes

After anesthetized, C57BL/6 mouse primary hepatocytes had been isolated by a two-step collagenase digestion method (Meng et al., 2015a). The isolated hepatocytes were collected and resuspended with William’s E medium (Invitrogen, Carlsbad, CA, USA) with penethamate (100 U/mL), streptomycin (100 U/mL), dexamethasone (0.1 mM), and thyroxine (1 mM). Then, hepatocytes were seeded into collagen type I (Sigma, St. Louis, MO, USA) pre-coated cell culture dishes. After 24 h incubation, hepatocytes were coated with collagen I solution and then cultivated in complete medium to achieve sandwich culture model (Li et al., 2016).

### RNA Silencing Experiments

Mouse primary hepatocytes had been transiently transfected with siRNA which targeting mouse HNF1α (sc-35568) or a negative control siRNA utilizing Lipofectamine^TM^ 3000 (Invitrogen, Carlsbad, CA, USA). Seven hours later, medium had been changed with fresh medium. After 48 h, DMSO or SRT1720 (10 μM) had been added to the cell culture medium for 12 h. Then, primary hepatocytes cells were collected for further QPCR experiment.

### Liver Enzymes Assays

Mouse livers had been homogeneous with ice-cold 0.9% normal saline and the slurry was centrifuged at 4°C (3500 rpm/min), then supernatant had been utilized for hepatic enzymes assays. The levels of IL-6, TNF-α, and myeloperoxidase (MPO) activity in supernatant had been measured to evaluate inflammatory reactions and neutrophil activation in livers. The IL-6 and TNF-α levels were measured by ELISA kits (UNIV-bio, Shanghai, China). MPO activity was measured by a commercial assay kit (Jian cheng Bio-engineering Institution, Nanjing, China). The activities of entire antioxidant capacity (T-AOC), glutathione (GSH), malondialdehyde (MDA), and superoxide dismutase (SOD) had been determined to access the oxidant state in the hepatic constitution. Liver T-AOC, GSH, SOD, and MDA activities had been measured by their respective assay kit (Jian cheng Bioengineering Institute, Nanjing, China).

### Statistical Analysis

Statistics are processed with a software called Graphpad Prism (5th version) (Graph-Pad, La Jolla, CA, USA) and are shown as the mean ± SD. Through utilizing one-way variance analysis, the significance of the statistic from multiple groups is determined. The data is considered statistically significant when *P* < 0.05.

## Results

### Protective Effects of SRT1720 on Cholestasis and Hepatotoxicity Induced by ANIT

**FIGURE 1:**
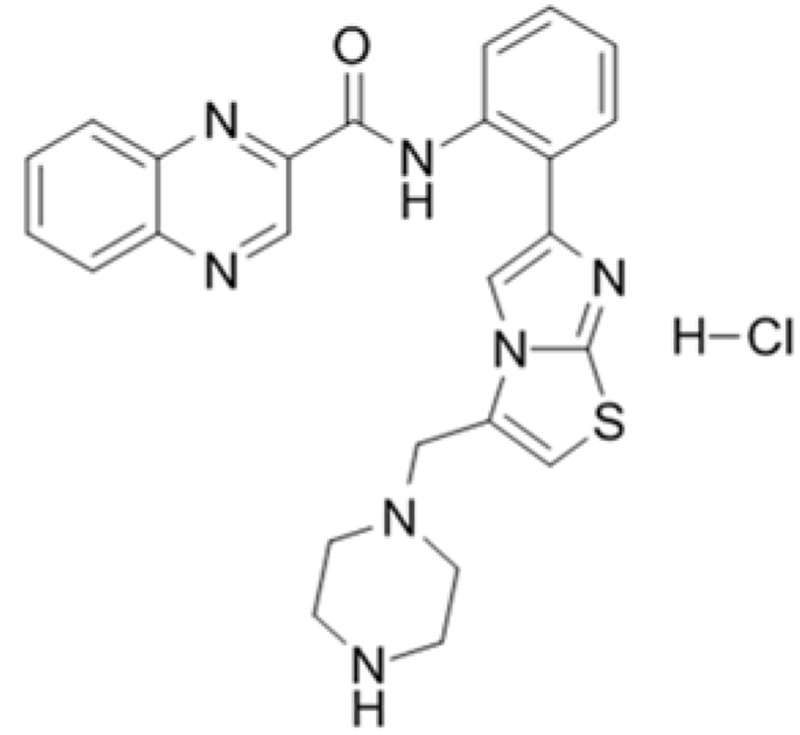
**The chemical structure of SRT1720**.

**FIGURE 2:**
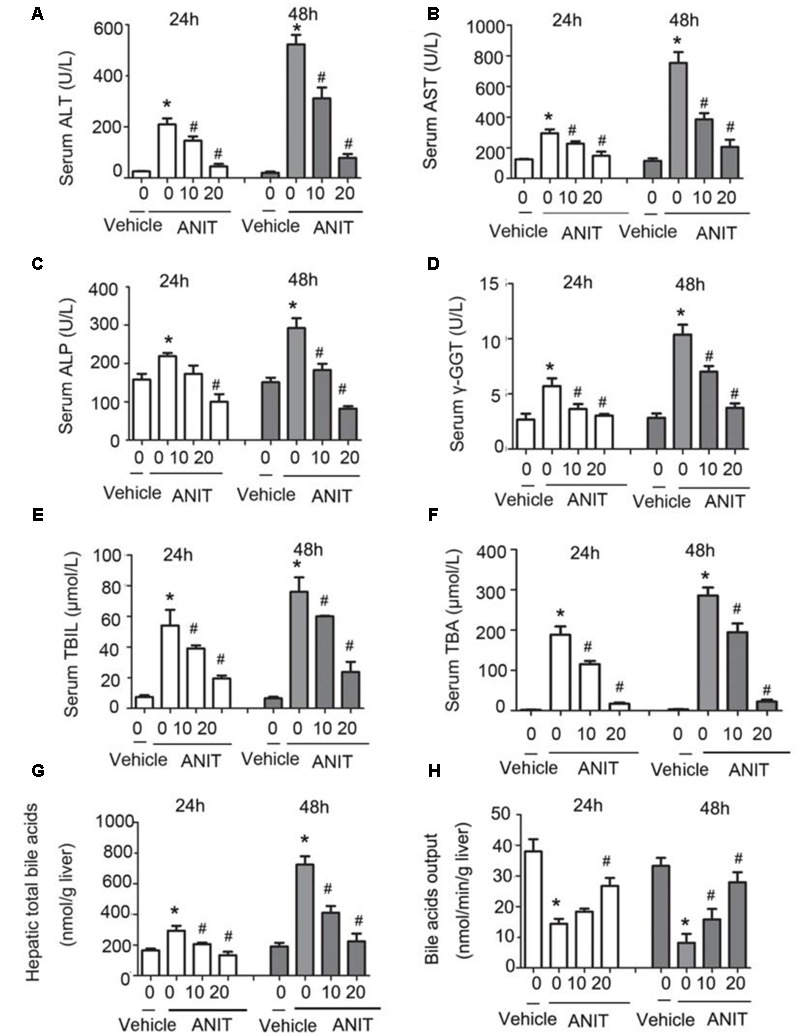
**Protective effects of SRT1720 on cholestasis and hepatotoxicity induced by alpha-naphthylisothiocyanate (ANIT).** Biochemical indicators in mice treated with vehicle or, 10 or, 20 mg/kg of SRT1720, were determined at the time points of 24 and 48 h after ANIT or vehicle administration. **(A)** Serum ALT, **(B)** AST, **(C)** ALP, and **(D)** γ-GGT activity, as well as **(E)** serum total bilirubin, **(F)** serum total bile acid (TBA), and **(G)** hepatic TBA. **(H)** Bile acid output decreased in mice due to ANIT and was significantly ameliorated in SRT1720-treated mice. Data are the mean ± SD (*n* = 6). ^∗^*P* < 0.05 versus vehicle; ^#^*P* < 0.05 versus vehicle +ANIT.

**FIGURE 3:**
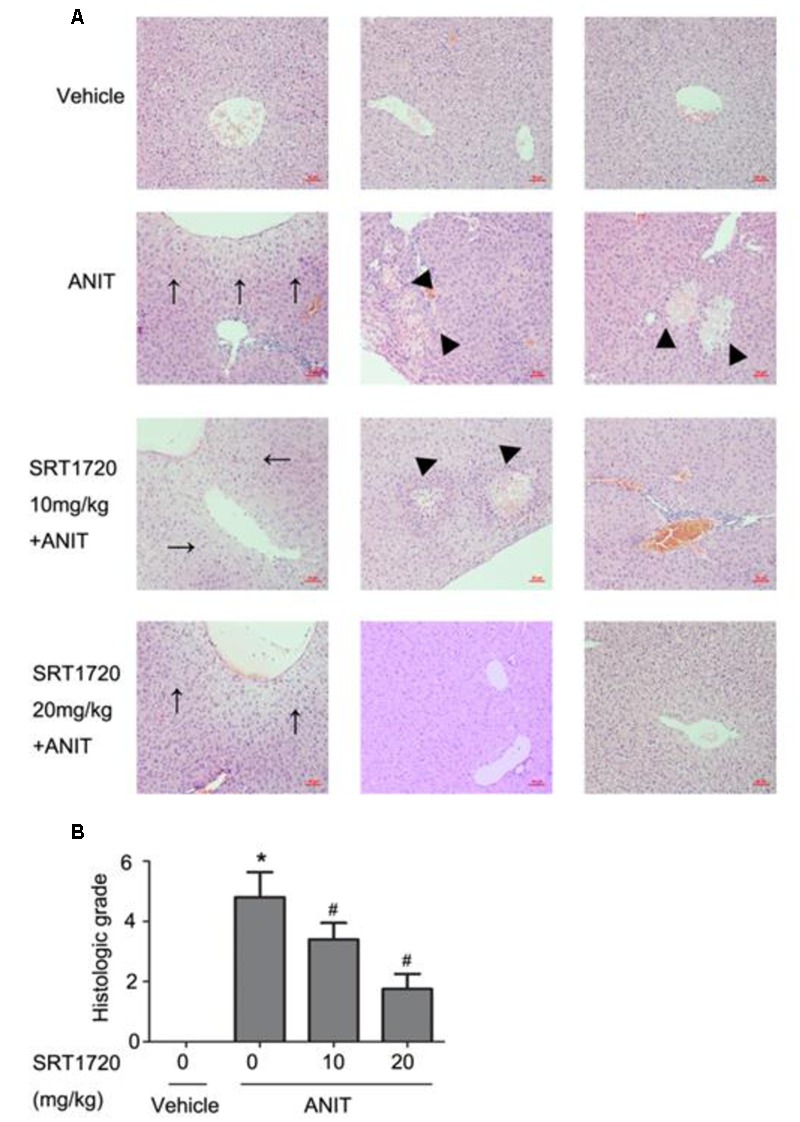
**SRT1720 attenuated liver injury induced by ANIT in mice.** Images of H&E stained liver sections (200× magnification) at 48 h after ANIT administration were shown. **(A)** Vehicle: no histological change was observed; ANIT: necrotic and degenerative changes were observed. SRT1720 10mg/kg+ANIT: small necrotic and degenerative changes were observed; SRT1720 20 mg/kg+ANIT: small degenerative changes were observed. Areas of severe liver necrosis were marked by triangle arrows, and degenerative changes were marked by normal arrows. **(B)** Graph showed the quantitative analysis of necrotic lesions and degenerative changes. Data are the mean ± SD (*n* = 6). ^∗^*P* < 0.05 versus vehicle; ^#^*P* < 0.05 versus vehicle +ANIT.

To determine whether SRT1720 could reverse mice cholestatic liver injury induced by ANIT, C57BL/6 mice were treated with ANIT, 0.2% CMC-Na, or SRT1720 (10 mg/kg or 20 mg/kg) for 5 days. The SRT1720 chemical structure was showed in **Figure 1**. Biochemical indicators in mice intraperitoneal administered vehicle, with 10 or 20 mg/kg of SRT1720, were evaluated 24 or 48 h later after taking ANIT. Taking ANIT led to the damage in the liver which was demonstrated by the remarkable increase in the serum AST and ALT expressions compared with control group. Upon SRT1720 treatment, plasma AST and ALT levels remarkably lessened by 70% compared with ANIT-treated group (**Figures 2A,B**). ALP is produced by osteoblasts, absorbed by the liver, and then secreted through the bile acid. The level of plasma ALP will increase significantly during cholestasis. The γ-GGT is mainly distributed in the liver and intrahepatic bile duct epithelium, when cholestasis, this enzyme may countercurrent into the blood. ANIT administration remarkably increased the plasma levels of ALP and γ-GGT and SRT1720 remarkably reduced them compared to ANIT-treated group (**Figures 2C–D**). Furthermore, SRT1720 dosage dependently reversed ANIT-induced increases in, TBA total bilirubin and hepatic TBA (**Figures 2E–G**). ANIT reduced bile acid output at 24 h after administration, and SRT1720 remarkably attenuated this variation (**Figure 2H**). Because all of the biochemical indicators of hepatobiliary injury were observed to be higher at 48 h than 24 h after ANIT administration, the 48 h time point had been selected for the subsequent studies. The H&E staining results demonstrated that SRT1720 treatment remarkably attenuated ANIT-induced degenerative changes and necrotic foci in the livers (**Figure 3**), which further demonstrated protective effects of SRT1720 on liver damage which induced by ANIT. In conclusion, all these results above demonstrated that SRT1720 could provide remarkable protective effects on cholestasis and hepatotoxicity induced by ANIT.

### SRT1720 Altered mRNA Levels of Bile Acid Transporters

**FIGURE 4:**
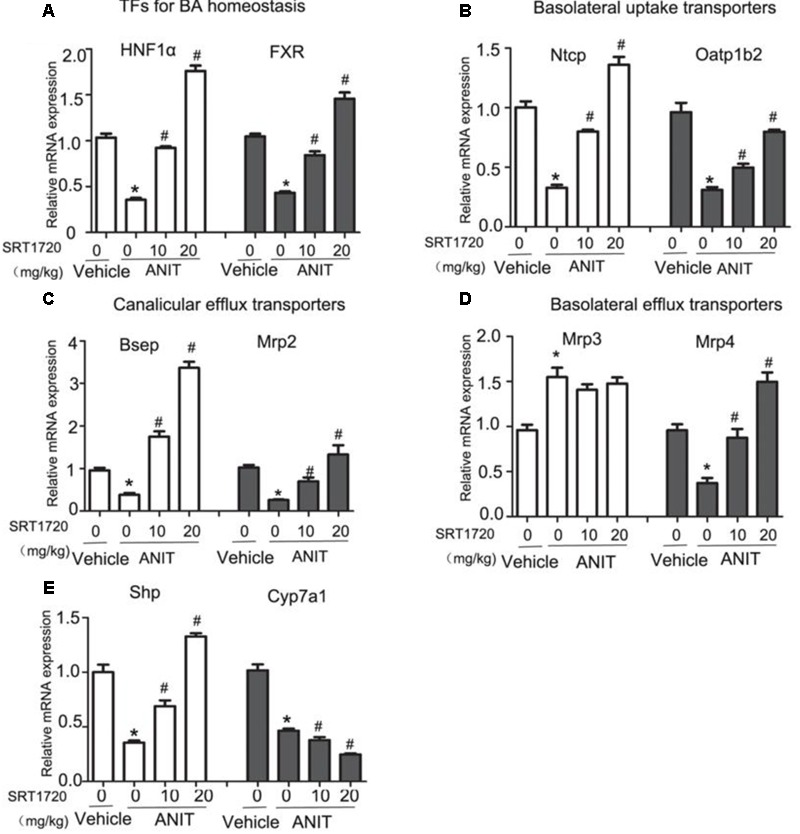
**SRT1720 altered the gene expressions of hepatic transporters involved in bile acid transport in mice total livers.** Quantitative real-time PCR analysis was performed to measure the gene expression levels **(A)** HNF1α and FXR, **(B)** Ntcp and Oatp1b2, **(C)** Bsep and Mrp2, **(D)** Mrp3 and Mrp4, **(E)** Cyp7A1 and Shp. Data are the mean ± SD (*n* = 6). ^∗^*P* < 0.05 versus vehicle; ^#^*P* < 0.05 versus vehicle +ANIT.

To explain the potential mechanisms of SRT1720 hepatoprotective effects, the gene expressions of bile acid transporters had been evaluated by real-time PCR. First, we determined the expressions of HNF1α and FXR, both of which are important nuclear receptors to regulate systemic bile acid metabolism. The gene expressions of HNF1α and FXR were reduced by 65 and 56%, respectively, following ANIT administration. SRT1720 treatment increased the mRNA levels of HNF1α and FXR (**Figure 4A**). Just like illustrated in **Figure 4B**, ANIT caused a decrease in the gene expressions of Ntcp and Oatp1b2, and SRT1720 treatment enhanced both of them. Then, we examined the gene expressions of Bsep and Mrp2. **Figure 4C** showed that ANIT reduced the expressions of Bsep and Mrp2 remarkably and SRT1720 treatment increased their expressions. **Figure 4D** illustrated that the mRNA level of Mrp3 showed a 1.5-fold increase, while SRT1720 demonstrated no effect at all. The mRNA expression of Mrp4 was reduced by ANIT, and SRT1720 increased its gene expression. Together, the above results suggested that ANIT remarkably decreased the expressions of HNF1α, FXR, Ntcp, Oatp1b2, Bsep, Mrp2, and Mrp4, which aligns with the findings of a previous research (Wang T. et al., 2014). SRT1720 treatment restored the expressions of HNF1α, FXR, Ntcp, Oatp1b2, Bsep, Mrp2, and Mrp4, which caused an increase of bile acid efflux and influx in the liver. However, SRT1720 did not have remarkable effect on Mrp3 expression.

### SRT1720 Altered the mRNA Level of Cyp7a1

In addition to the transporters mentioned before, bile acid synthetic enzymes are also included in bile acid homeostasis. In order to further analyze the hepatoprotective effects of SRT1720, we examined the mRNA expression of Cyp7a1, the enzyme in bile acid synthesis that limits the reaction rate. As illustrated in **Figure 4E**, the mRNA level of Cyp7a1 was decreased in ANIT group compared with control group. And SRT1720 therapy further decreased mRNA level of Cyp7a1. We then examined the mRNA level of Shp which is its upstream gene. Shp activation has been demonstrated to restrain Cyp7a1 transcriptionally (Kong et al., 2012b). SRT1720 treatment increased the mRNA level of Shp (**Figure 4E**). These findings suggested that the inhibition of Cyp7a1 by SRT1720 could be mediated by FXR-Shp signaling pathway.

### SRT1720 Regulated Bile Acid Detoxifying Enzymes

**FIGURE 5:**
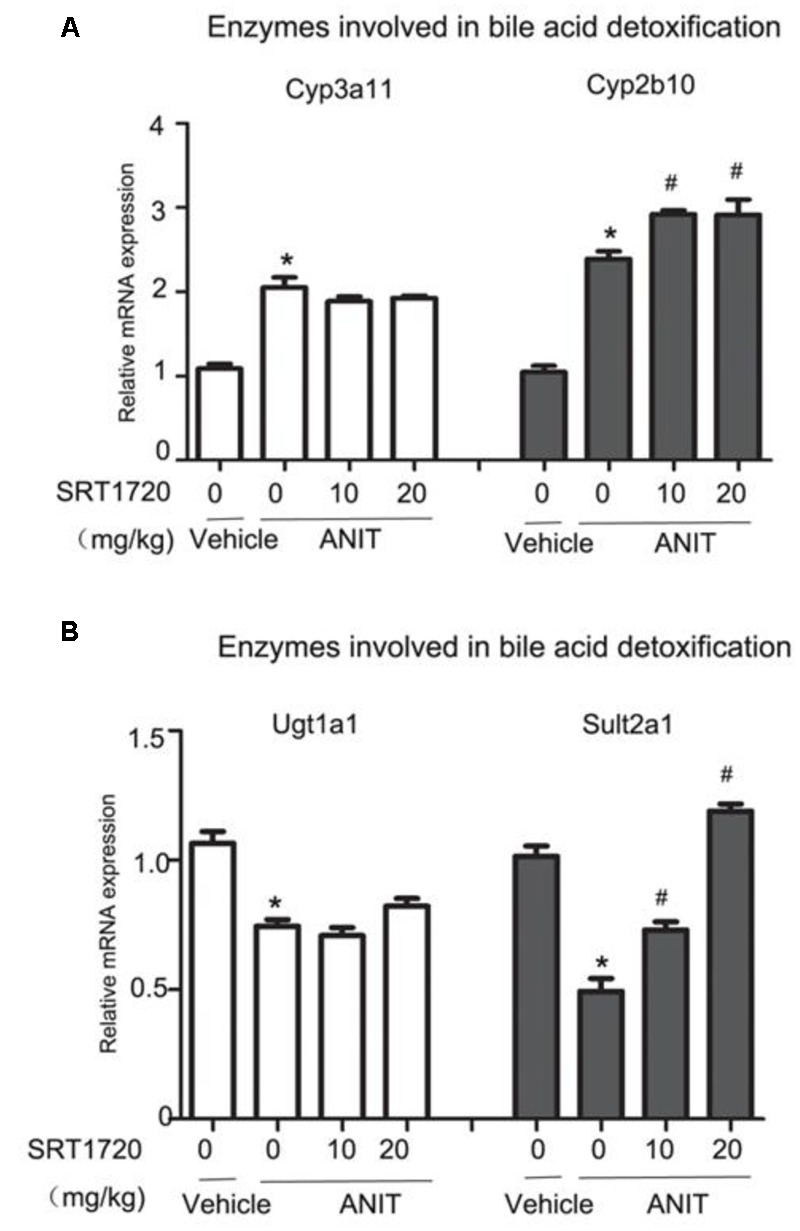
**SRT1720 altered the gene expressions of hepatic enzymes involved in bile acid metabolism in mice total livers. (A)** Bile acid metabolizing enzymes, including the phase I enzymes Cyp3a11 and Cyp2b10 were shown. **(B)** Bile acid metabolizing enzymes, including the phase II enzymes Ugt1a1 and Sult2a1, were shown. Data are the mean ± SD (*n* = 6). ^∗^*P* < 0.05 versus vehicle; ^#^*P* < 0.05 versus vehicle +ANIT.

Phase I enzymes like Cyp2b10 and Cyp3a11, and phase II enzymes like UDP-glucuronosyltransferase 1a1 (Ugt1a1) and sulfotransferase 2a1 (Sult2a1) are bile acid detoxifying enzymes in the liver. As shown in **Figure 5A**, ANIT increased the mRNA levels of Cyp3a11 and Cyp2b10, and SRT1720 increased Cyp2b10 mRNA level further. ANIT decreased mRNA levels of Sult2a1 and Ugt1a1, and SRT1720 caused an increase in the mRNA expression of Sult2a1 (**Figure 5B**). These results indicated that mRNA levels of bile acid detoxifying enzymes could be enhanced by SRT1720 compared with ANIT group.

### SRT1720 Up-regulated the Protein Levels of Bsep, Mrp2, and FXR in Mice

**FIGURE 6:**
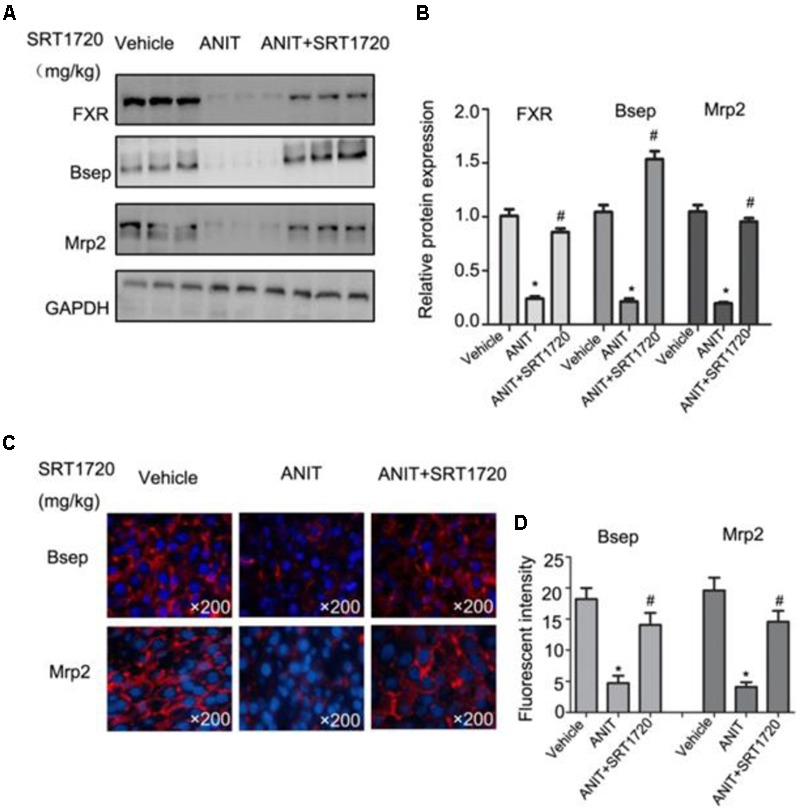
**SRT1720 restored the protein expressions of FXR, Bsep, and Mrp2 in mice total livers. (A)** Western blot analysis was used to measure FXR, Bsep, and Mrp2 expressions. **(B)** Specific band intensity was quantified, normalized to GAPDH. **(C)** Immunofluorescence staining of frozen liver sections showing Bsep and Mrp2 expressions. **(D)** Fluorescent intensities of Bsep and Mrp2 were measured by Image-Pro Plus software.

To check Real-time PCR results, western blotting method was used to evaluate the protein expressions of Bsep, Mrp2, and FXR. **Figures 6A,B** indicated that ANIT caused decreases of protein expressions of Bsep, Mrp2, and FXR, and a 20 mg/kg SRT1720 treatment caused an increase in these levels. **Figures 6C,D** showed that fluorescence intensities of Bsep and Mrp2 in cytomembranes were remarkably decreased by ANIT, which suggested that bile acid transporters were impaired by ANIT. SRT1720 increased fluorescence intensities of Bsep and Mrp2 in cytomembranes remarkable.

### SRT1720 Up-regulated the Expressions of FXR Target Genes *In Vitro*

**FIGURE 7:**
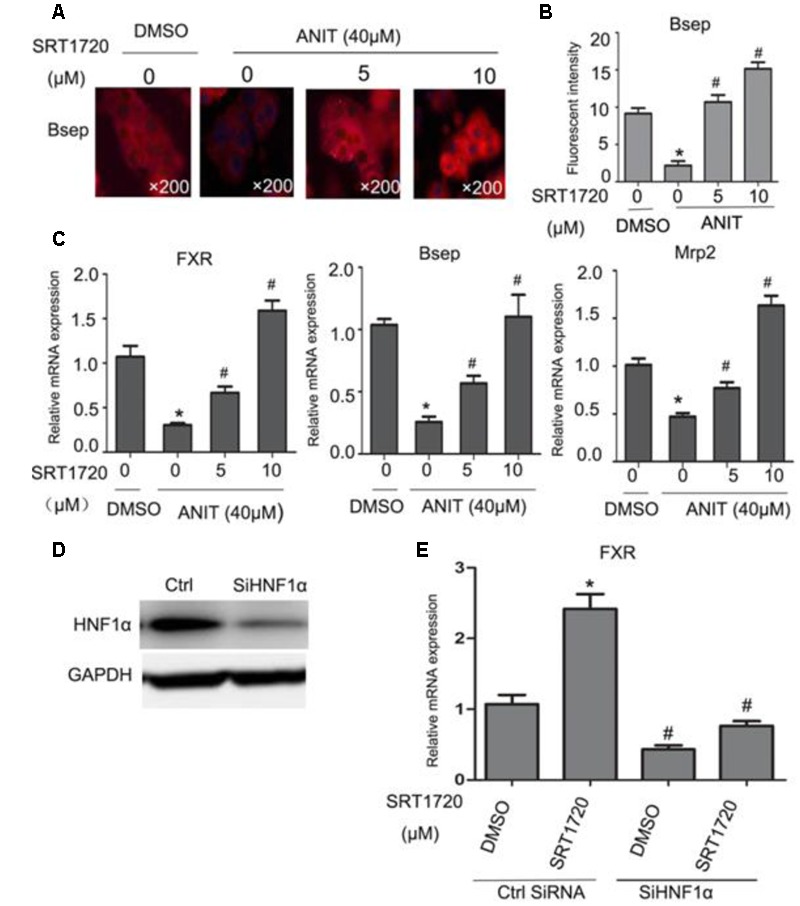
**Effects of SRT1720 on FXR, Bsep, and Mrp2 expressions *in vitro*. (A)** Immunofluorescence was used to investigate the effects of SRT1720 on Bsep; after treatment with ANIT for 24 h, Bsep decreased in fluorescence intensity, and SRT1720 (5 μM, 10 μM) induced up-regulation of Bsep, which was observed compared with groups treated with ANIT. **(B)** Fluorescent intensity of Bsep was measured by Image-Pro Plus software. **(C)** Mouse primary hepatocytes were treated with ANIT (40 μM), and SRT1720 (5 μM, 10 μM); ANIT inhibited FXR, Bsep, and Mrp2 expression at the mRNA level, and SRT1720 increased all of them. **(D)** HNF1α silencing efficiency was measured by Western blot. **(E)** HNF1α silencing abrogated the regulation of FXR by SRT1720 (10 μM) in mice primary hepatocytes. ^∗^*P* < 0.05 versus DMSO alone; ^#^*P* < 0.05 versus SRT1720 alone.

To determine whether SRT1720 could regulate the expressions of FXR and its downstream genes *in vitro*, FXR, Bsep and Mrp2 mRNA levels were quantified in mouse primary hepatocytes which treated with different concentrations of SRT1720. SRT1720 was incubated with ANIT for 24 h, as shown in **Figures 7A,B**, in control cells, Bsep staining was localized in thin continuous lines outlining the cells, and after treatment with ANIT for 24 h, the fluorescence intensity of BSEP was decreased, and SRT1720 increased Bsep fluorescence intensity compared with the cells treated with ANIT, which suggested that the inhibitory effects of ANIT could be improved by SRT1720. As shown in **Figure 7C**, SRT1720 was incubated with ANIT for 12 h, ANIT inhibited FXR, Bsep and Mrp2 expressions at the mRNA level, and SRT1720 increased all of them. To determine the potential mechanism of SRT1720 mediated activation of FXR, HNF1α gene silencing experiment was performed *in vitro*. The protein expression of HNF1α had been evaluated by western blot analysis after transfected with HNF1α siRNA and a control siRNA (**Figure 7D**). **Figure 7E** revealed that SRT1720 increased the mRNA level of FXR in normal mouse primary hepatocytes and it had no effect on it in HNF1α deficiency mouse primary hepatocytes (**Figure 7E**). These results demonstrated that SRT1720 mediated activation of FXR was largely through HNF1α.

### Effects of SRT1720 on Hepatic MDA, GSH, T-AOC, and SOD Activities

**FIGURE 8:**
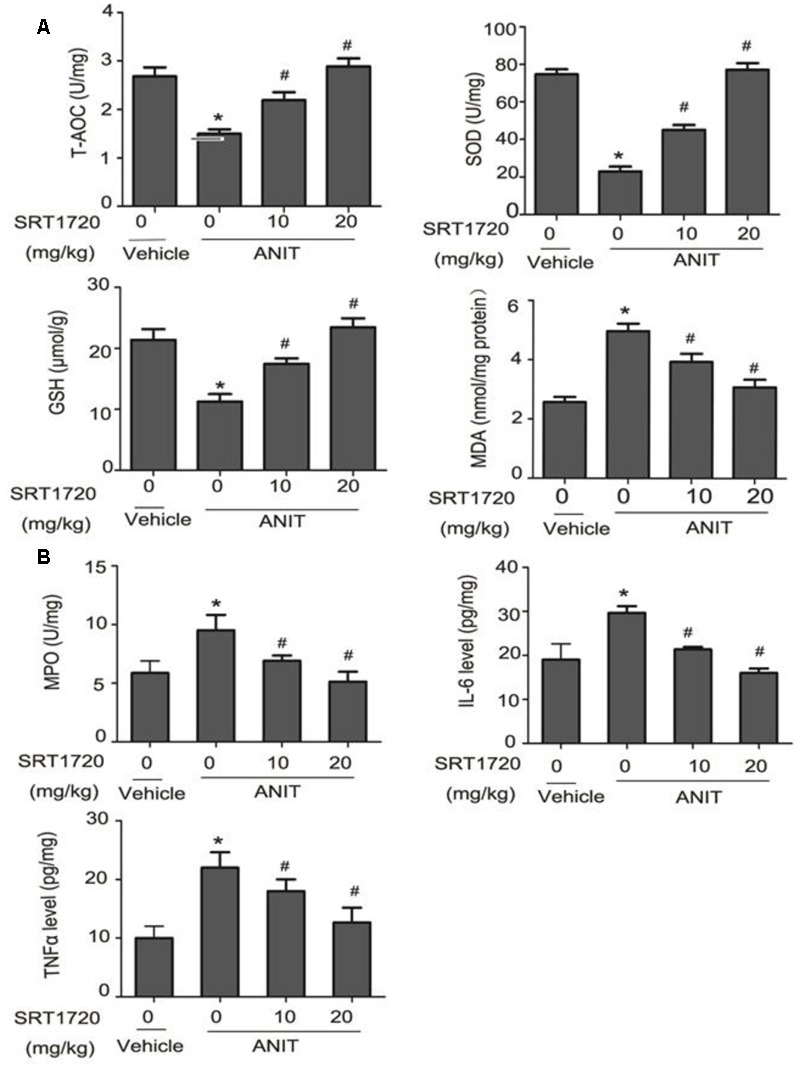
**Effects of SRT1720 on antioxidant system and inflammatory factors *in vivo*. (A)** Effects of SRT1720 on hepatic T-AOC, SOD, GSH, and MDA activities at 48 h after ANIT administration in mice. **(B)** Effects of SRT1720 on MPO activity, TNF-α and IL-6 levels at 48 h after ANIT administration in mice. Data are the mean ± SD (*n* = 6). ^∗^*P* < 0.05 versus vehicle; ^#^*P* < 0.05 versus vehicle +ANIT.

**Figure 8A** revealed that the activities of hepatic T-AOC, SOD, and GSH in the livers were significantly inhibited by ANIT, which suggested that ANIT could cause antioxidant defense systems disruption in the liver. Comparatively, activities of T-AOC, SOD, and GSH could be restored by SRT1720 at 10 mg/kg and 20 mg/kg, respectively (**Figure 8A**). Forty-eight hours after ANIT-treatment, hepatic MDA level increased notably in the group with ANIT treatment compared with the control group. And both doses of SRT1720 significantly decreased MDA concentrations (**Figure 8A**). Taken together, SRT1720 exerted an antioxidant effect on ANIT-induced hepatotoxicity and cholestasis.

### Effects of SRT1720 on Hepatic Inflammatory Cytokines and MPO Activity

Forty-eight hours later, the hepatic MPO activity in the control group was lower than the group with ANIT treatment. And SRT1720 dose-dependently inhibited MPO activity that was induced by ANIT (**Figure 8B**). This was further confirmed by the Immunohistochemical staining of MPO (Supplementary Figure S1A). Multiple reports demonstrated that inflammatory reaction was related to cholestasis and SRT1720 could alleviate inflammatory reaction. ANIT increased the levels of TNF-α and IL-6 at 48 h and SRT1720 caused a decrease in the concentrations of TNF-α and IL-6. The results revealed that SRT1720 has an anti-inflammatory effect in ANIT-treated mice (**Figure 8B**).

### SRT1720 Up-regulated the Expressions of Nrf2 Target Genes

**FIGURE 9:**
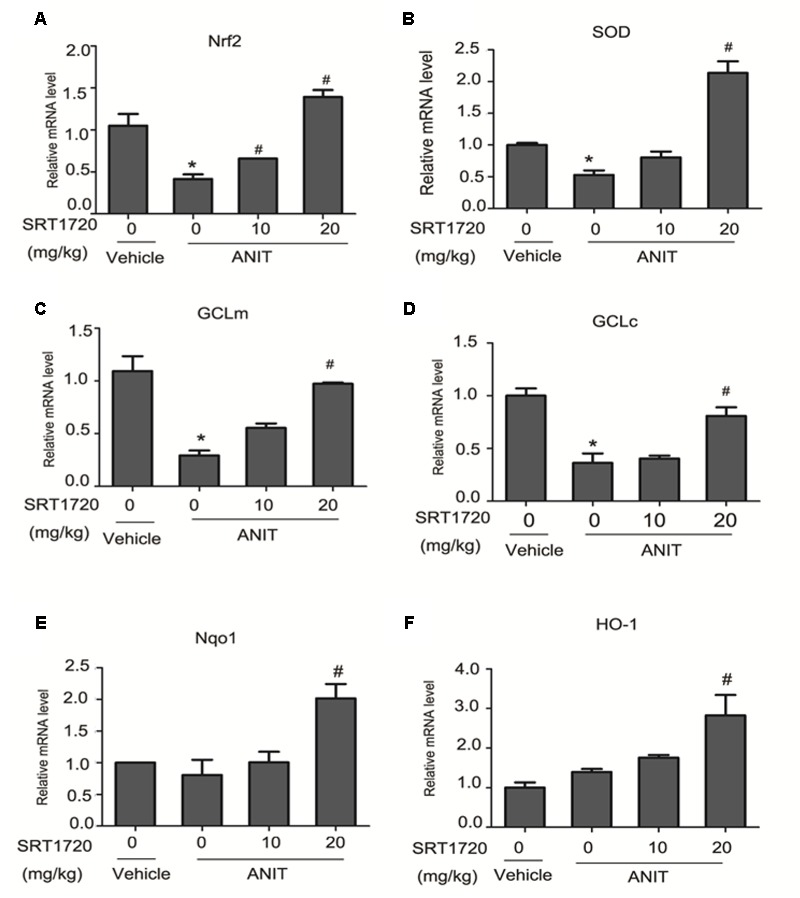
**SRT1720 altered gene expressions of Nrf2/ARE signalling in ANTI-induced liver hepatotoxicity and cholestasis in mice. (A)** Nrf2, **(B)** SOD, **(C)** GCLm, **(D)** GCLc, **(E)** Nqo1, and **(F)** HOO-1. Data are the mean ± SD (*n* = 6). ^∗^*P* < 0.05 versus vehicle; ^#^*P* < 0.05 versus vehicle +ANIT.

**Figure 9** revealed that mRNA levels of Nrf2 and its target genes were increased by SRT1720 in ANIT-treated group dose-dependently. The mRNA levels Nrf2, GCLc, GCLm, and SOD were significantly decreased by ANIT at 48 h and SRT1720 up-regulated the expressions of Nrf2 and its target genes dose-dependently (**Figures 9A–D**). ANIT did not inhibit expressions of HO-1 and Nqo1, and SRT1720 increased their mRNA levels dose-dependently (**Figures 9E,F**). Taken together, these results clearly demonstrated that SRT1720 exerted an anti-oxidation effect through activating the Nfr2/ARE pathway in ANIT-treated mice (**Figure 9**).

## Discussion

**FIGURE 10:**
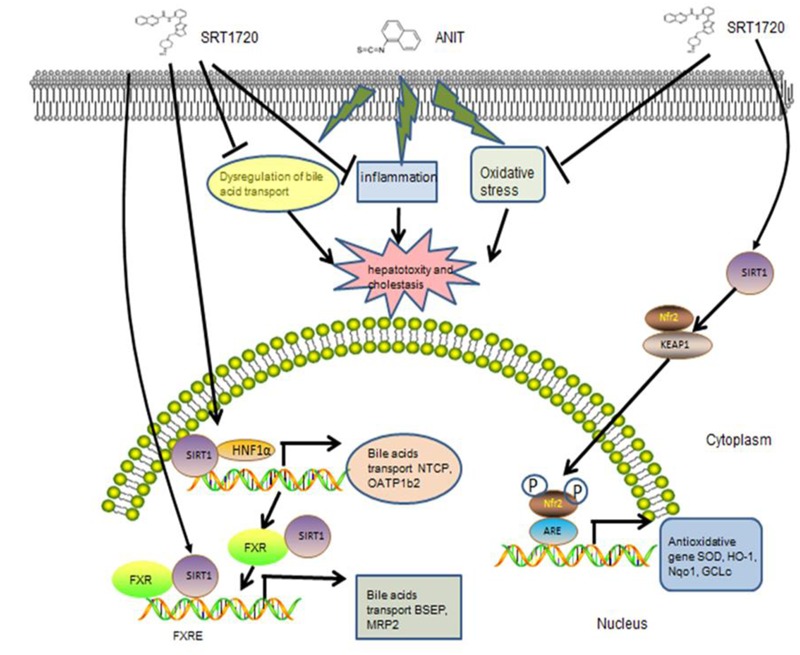
**The possible mechanism of SRT1720 attenuated ANIT-induced cholestasis and liver injury**.

Cholestasis, with the impairment of bile flow, could cause excessive bile acids accumulated in liver and could lead to liver cirrhosis and biliary fibrosis ultimately (Anwer, 2014). It is demonstrated that ANIT could lead to cholestasis through injuring biliary epithelial cell directly and inhibiting the expressions of bile acid transporters. It has been demonstrated that ANIT glutathione conjugate transported into bile through Mrp2. Mrp2 is an important hepatic canalicular efflux transporter that responsible for biliary excretion of various conjugated endobiotics and xenobiotic. The expression of Mrp2 in liver is different between male and female mice. Normally speaking, the expression of Mrp2 in male mice is lower than female mice. So, different regulation of Mrp2 expression and function between males and females may cause different liver damage during cholestasis (Kong et al., 2012a; **Figure 10**).

It is concluded that the reasons for liver injury caused by ANIT comprise the dysregulation of bile acid transporters, the release of inflammatory mediators and the hepatic antioxidant defense system that improves lipid peroxidation of liver (Ohta et al., 1999; Cui et al., 2009). Previous studies have reported that SRT1720 could reverses the liver injury induced by 1% cholic acid feeding of mice by hepatic and extra hepatic mechanisms (Kulkarni et al., 2016). In current study, our results clearly indicated that SRT1720 could alleviate cholestatic liver injury caused by ANIT according to the given dosage. Since the dosage of SRT1720 given to mice is 20 mg/kg and the dosage convert coefficient between mice and human is 0.11, so the dosage of SRT1720 for human is about 2.2 mg/kg. SRT1720 not only significantly increased the expressions of bile acid uptake transporters (Ntcp, Oatp1b2), but also increased the expressions of efflux transporters (Bsep, Mrp2). SRT1720 decreased the levels of inflammatory cytokines in the liver, leading to a reduction in the inflammatory process. Moreover, SRT1720 alleviated liver oxidative stress response via activation of Nrf2/ARE signal pathway.

SRT1720 caused a decrease in the elevations of serum ALT, ALP, AST, TBIL, TBA, and γ-GGT levels that are induced by ANIT (**Figure 2**). After SRT1720 medication, the histological injuries healed. (**Figures 3A,B**). These results indicated that SRT1720 has a significant effect on liver damage that is induced by ANIT. Various enzymes as well as transporters played important roles in bile acid homeostasis. Plenty of nuclear receptors are responsible for regulating these transporters as well as enzymes. Among them, HNF1α was called the ‘regulator of regulators’, and a recent study reported that the plasma TBA level in HNF1α^-/-^ mice was higher than normal mice. It was demonstrated that Ntcp and Oatp1b2 were direct target genes of HNF1α, and HNF1α^-/-^ mice showed decreased expressions of these genes, and thus caused damage to uptake of bile acid as well as increase in plasma’s concentration in bile acid (Shih et al., 2001). The transporters on basolateral domains, Ntcp and Oatp1b2, are responsible for reabsorption of bile acid from blood to hepatocyte. It was reported that bile acid could decrease the expression of HNF1α, Oatp1b2 and Ntcp (Jung and Kullak-Ublick, 2003). ANIT down-regulated expressions of Ntcp and Oatp1b2 in mouse liver which consistent with a previous study (Wang T. et al., 2014), and SRT1720 treatment increased ANIT-suppressed HNF1α, Oatp1b2, and Ntcp expressions. Two mechanisms might be involved in which SRT1720 can restore HNF1α gene expression: 1. SRT1720 increased HNF1α expression directly in ANIT-treated mice; or 2. SRT1720 decreased hepatic TBAs in ANIT-treated mice, and it alleviated bile acids-suppressed HNF1α, Ntcp, and Oatp1b2. How SRT1720 regulates the activity of HNF1α is unknown, and more experiments are needed further.

HNF1α could directly bind to the promoter of FXR to regulate its expression (Shih et al., 2001). It has been demonstrated that Bsep and Mrp2, two major canalicular bile acid transports, are FXR target genes. FXR induces not only Bsep and Mrp2 but also Shp expression, which reversely causes transcriptional repression of Cyp7a1 gene. Both serum and liver TBA levels were increased in FXR^-/-^mice compared with normal mice (Lu et al., 2012). The paper has shown that ANIT has led to significant decrease in the gene expressions of FXR, Bsep and Mrp2, whereas SRT1720 caused an increase in all of them. Cyp7a1 is the rate-limiting enzyme in bile acids synthesis which also important in bile acid metabolism. The mRNA level of Cyp7a1 was decreased by ANIT and SRT1720 treatment further decreased ANIT-suppressed Cyp7a1 expression. Since Cyp7a1 limits the rate of cholesterol convert to bile acid, the inhibition of Cyp7a1 may increases the content of cholesterol in the liver (Ferrell et al., 2016). In the liver, BA hydroxylation and hydrophobicity are regulated by phase I enzymes like Cyp2b10 as well as Cyp3a11, and phase II enzymes like Ugt1a1 and Sult2a1 (Meng et al., 2015b). Cyp3a11 is a target gene of pregnane X receptor (PXR), and Cyp2b10 is a target gene of constitutive androstane receptor (CAR) (Lee et al., 2015) Supplementary Figure S2. Sult2a1 is the target gene of FXR (Meng et al., 2015b). However, SRT1720 increased the gene expressions of Cyp2b10 and Sult2a1 in mice, suggesting that CAR may also involve in hepatoprotection of SRT1720 (**Figure 5**). In addition to HNF1α and FXR, there are many others nuclear receptors that also participate in bile acids metabolism, such as CAR, PPARα, and SRT1720 may also affect them to alleviate ANIT-induced cholestasis; thus, additional experiments are needed to determine its mechanism.

To further analyze the influence of SRT1720 on FXR signaling, Bsep, Mrp2, and FXR expressions were quantified in sandwich cultured mouse hepatocytes that treated with different dose of SRT1720. The evidence demonstrated that SRT1720 increased the expressions of FXR, Mrp2 and Bsep (**Figures 7A,B**). As shown in **Figure 7C**, the activation of FXR induced by SRT1720 was abrogated after HNF1α silencing (**Figure 7D**).

It was commonly recognized that oxidative stress also involved in cholestatic liver injury caused by ANIT (Aboutwerat et al., 2003). ROS is an oxidants which produced by almost all cells. Excessive ROS can injure cells and tissues. SOD is an important antioxidant enzyme which can prevent damages caused by oxidants. It promotes the production of O_2_ and H_2_O_2_ from O_2_^-^ to prevent the initiation of free-radical chain reactions (Ohta et al., 1999). T-AOC is considered as a vital indicator of antioxidant enzyme system for removing excessive ROS from the cells. GSH is one of the most important cellular antioxidants which can eliminate ROS (Lu, 2009). MDA is the product which produced by the interaction between ROS and polyunsaturated fatty acids. It was used as an indicator of oxidative stress. Assessing the level of MDA is a reliable method for assessing the degree of oxidative damage to the cell membrane (Duval et al., 1990). Significantly higher levels of MDA and reduced activities of GSH, T-AOC and SOD in hepatic tissue were observed in the present study, indicating antioxidant defenses were decreased by ANIT and oxidative damage were existed in the liver. SRT1720 markedly restored SOD, GSH and T-AOC activities (**Figure 8A**). It is well known that developments of hepatic injuries caused by ANIT are largely influenced by infiltration of neutrophils. The indicator of neutrophils is MPO enzyme. In the current research, ANIT caused a significant increase in the MPO activity in the liver which indicated infiltration of neutrophil. The amount of neutrophil that infiltrated in the liver was reduced by SRT1720. In addition, it is observed that SRT1720 decreased liver TNF-α and IL-6 levels. So, we considered that SRT1720 could decrease neutrophil recruitment and the release of inflammatory cytokines to alleviate liver injury caused by ANIT (**Figure 8B**).

Nrf2 is a vital transcription factor which can regulate various antioxidative stress genes (Okada et al., 2009). Nrf2 activation could alleviate cholestasis caused by BDL and ANIT models through activation of many antioxidative stress genes (Aleksunes et al., 2006; Tanaka et al., 2009). Several studies have reported that Nrf2 is suppressed by actin-binding protein keap1 (Kelch-like ECH-associated protein 1) in normal cells. Upon oxidative stress, Nrf2 dissociates from of Keap1 and then translocates into nucleus to bind antioxidant response element (ARE) in order to initiate the transcription of a series of antioxidative stress genes (Wang G. et al., 2014). It has been proved that Nrf2 can regulate the expressions of GCL subunits expressions (GCLm and GCLc), SOD, HO-1 and Nqo1 (Arisawa et al., 2009). The paper revealed that the mRNA levels of Nrf2, SOD, GCLc, and GCLm in the group treated with ANIT decreased significantly compared with control group (**Figure 9A**) and SRT1720 increased all of them. However, the gene expressions of Nqo1 and HO-1 were not affected by ANIT. SRT1720 also increased their mRNA levels. Thus, sufficient evidence proved that SRT1720 increases the mRNA levels of Nrf2 and its target genes in cholestasis which induced by ANIT.

## Conclusion

The current research demonstrated how SRT1720 exert its protective effect for liver damage and cholestasis induced by ANIT. And it suggested that activators of SIRT1 are effective agents for the treatment of cholestasis. Moreover, SIRT1 may be an effective therapeutic target for treating diseases related to cholestasis.

## Author Contributions

LY contributed to the acquisition of data. XL, ZY, XL, HY, ZY, and LS contributed to the writing of the manuscript. ZJ and LZ were in charge of the overall conception and design of the study.

## Conflict of Interest Statement

The authors declare that the research was conducted in the absence of any commercial or financial relationships that could be construed as a potential conflict of interest.
