# Supplementary material for: SRT1720 Alleviates ANIT-Induced Cholestasis in a Mouse Model
Source: Front Pharmacol. 2017 May 11;8:256. doi: 10.3389/fphar.2017.00256 (PMC5425580; doi:10.3389/fphar.2017.00256)
Supplement: Supplementary file 1 [file Data_Sheet_1.DOCX]

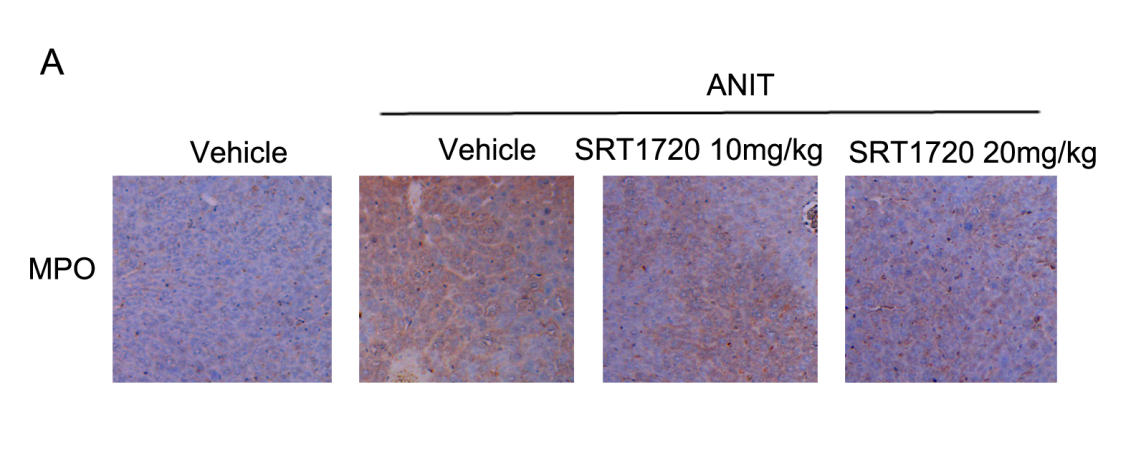


Supplementary Figure 1 | The Immunohistochemical staining results demonstrated that SRT1720 treatment significantly decreased ANIT-induced MPO in the livers of mice.


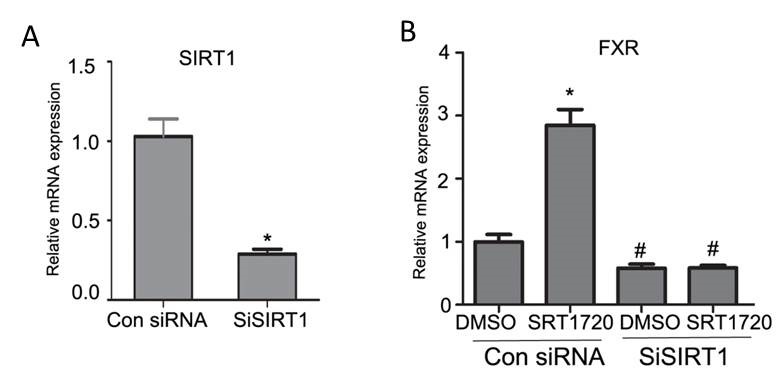


Supplementary Figure 2 **|** (A) SIRT1 silencing efficiency was measured by RT-PCR. (B) SIRT1 silencing abrogated the regulation of FXR by SRT1720 (10μM) in mice primary hepatocytes. **P*<0.05 versus DMSO alone; ^#^*P*<0.05 versus SRT1720 alone.
